# PREDICTING FUNCTIONAL DECLINE IN OLDER ADULTS: MORE ACTIVITY NOW EQUALS LESS DECLINE LATER

**DOI:** 10.1093/geroni/igz038.1912

**Published:** 2019-11-08

**Authors:** Brenda Whitehead

**Affiliations:** University of Michigan-Dearborn, Dearborn, Michigan, United States

## Abstract

According to the Function Spiral Model (Whitehead, 2017), aging attitudes influence activity engagement, which impacts functional ability via physical conditioning (or deconditioning). This study tests the activity ♢ conditioning ♢ function segment of the model using 59 older adults aged 61-92 (Mage = 76 at Time 1) who participated in 2 in-person assessments of physical health, gait, and function, spaced 3 years apart. Participants also completed mail-in questionnaires, reporting engagement in activities (walking, gardening, household chores, social clubs, etc.) at each time point. Hypotheses were 1) a lower activity level at Time 1 would predict greater decline in physical function across the 3-year span, and 2) that this effect would be mediated by changes in physical conditioning. Dependent t-tests revealed that both physical function—as indicated by the timed Get Up and Go test—and physical conditioning—as indicated by peak respiratory flow—declined during the period. The regression model testing the effect of activity engagement at Time 1 on decline in physical function (controlling for age, baseline function, and activity change) supported hypothesis 1 (-0.43, p = .003): more activity at Time 1 predicted less decline in physical function over time. Instead of supporting the mediation hypothesis, the model including both activity and conditioning demonstrated the strength of the activity at Time 1 effect, which actually increased in magnitude (-0.48, p = .001). Although the hypothesized mediation was not supported, the findings highlight current activity engagement as an important mechanism for slowing the progression of future age-related functional decline.

